# Keloids and Ultrasound Detected Fibroids in Young African American Women

**DOI:** 10.1371/journal.pone.0084737

**Published:** 2013-12-27

**Authors:** Quaker E. Harmon, Shannon K. Laughlin, Donna D. Baird

**Affiliations:** 1 Epidemiology Branch, National Institute of Environmental Health Sciences, National Institutes of Health, Research Triangle Park, North Carolina, United States of America; 2 Department of Obstetrics and Gynecology, Mayo Clinic, Rochester, Minnesota, United States of America; University of Louisville, United States of America

## Abstract

**Objective:**

Keloids and fibroids share a number of biologic and demographic similarities however there are no published reports of the association between them. The objective of this study was to investigate the association between self-reported keloids and ultrasound detected fibroids in a population of young African American women.

**Study Design:**

The Study of Environment, Life-style & Fibroids (SELF), is a volunteer cohort of over 1600 African American women aged 23-34 years recruited in Detroit, Michigan. Enrollment occurred between December 2010 and December 2012. Data are available for the first 1196 participants. Participants self-reported a history of raised (hypertrophic) scars or scars extending beyond the limits of the original injury (keloid) and had an enrollment pelvic ultrasound examination to detect prevalent fibroids. Log linear regression was used to model the association between abnormal scars and prevalent fibroids controlling for possible covariates. Among women with fibroids, associations between particular fibroid characteristics (tumor location, size or number) and scarring were assessed using chi-square and Mann Whitney U-tests.

**Results:**

Both abnormal scarring (keloids, 9.0%; hypertrophic scars, 28.3%) and fibroids (23.3%) were common in this cohort. There was no indication [adjusted Risk Ratio (95% Confidence Interval): 0.7 (0.5-1.1)] of an association between self-reported keloids and prevalent fibroids. Nor was there any association with hypertrophic scars. Specific characteristics of the prevalent fibroids were not associated with abnormal scarring.

**Conclusion:**

Despite similarly dysregulated extracellular matrices in keloids and fibroids, these conditions did not tend to co-occur in this young African American population.

## Introduction

Fibroids are common among African American women with an estimated cumulative incidence of 60% by age 40 and 80% by age 50[1]. In addition to significant symptoms which impact quality of life, treatment of fibroids results in hysterectomy for an estimated 20% of African American women. Available data suggests that fibroids develop at an earlier age (10-15 years earlier) in African Americans compared to Whites[1]. While numerous risk factors have been identified for fibroids, the pathogenesis of fibroids remain elusive[1]. One hypothesis[2,3] suggests that uterine injury may result in abnormal healing processes that initiate the formation of fibroids similar to the disordered healing seen in keloids or hypertrophic scars.

Keloids and hypertrophic scars develop following injury to the skin[4]. The resultant scars are raised and, in the case of keloids, extend beyond the initial margins of the original wound. While hypertrophic scars usually develop immediately following injury, keloids may develop at the site of an old injury and often recur following surgical treatment[5]. Although keloids and hypertrophic scars are considered distinct clinical phenotypes[4-6], pathology reports of surgically removed keloids[7] suggest that approximately 25% of clinically identified keloids lack the histologic characteristics of keloids and may be hypertrophic or normal scar tissue instead. In addition, some evidence is emerging that hypertrophic scars and keloids are simply different stages of a single phenotype[8].

While injury to the skin is required for keloid formation, there are few other known risk factors. A genetic predisposition to keloids has been suggested based on familial clusters and twin studies[9]. While women are not more prone to keloids than men, ovarian hormones may play a role. The incidence of keloids is highest between menarche and menopause and there is some suggestion that pregnancy may be a time of increased risk for keloid formation or growth[10]. However, a direct role of pregnancy related hormones has not been demonstrated. 

Keloids and fibroids share a number of clinical factors which suggest possible common etiologies. Both keloids and fibroids are more common in African Americans than Whites. Both also are composed of fibrotic tissues with excess extracellular matrix. Comparison of the components of the extracellular matrix in fibroids and keloids demonstrates similar composition of the proteoglycans, which differs from that of the respective healthy tissues[11,12]. Histologically the extracellular matrix composition is also similar with the collagen fibrils showing a tangled, rather than ordered [3,12], appearance. Molecular studies have also revealed a number of similarities in the genetic and protein profiles of these outcomes. Gene expression microarrays have described similar molecular environments [13] and differential expression of genes related to extracellular matrix[3]. Overexpression of transforming growth factor beta (TGFβ) has also been observed in both keloids and fibroids when compared to normal surrounding tissue[3]. The similarities at the molecular level suggest that although they arise from very different tissue types, these outcomes may share common pathogenic mechanisms or susceptibility factors.

While *in vitro* studies will continue to elucidate the pathogenesis of keloids and fibroids, epidemiologic studies can begin to address the co-occurrence of these two conditions. If keloids and fibroids do in fact share similar genetic susceptibility or pathophysiology, women with a history of keloids may have an increased risk of prevalent fibroids. Any association between these two outcomes would be most easily detected in African American women who are at high risk of both. The current study takes advantage of a prospective study of risk factor for fibroids among young African American women to examine the cross-sectional association between keloids and fibroids.

## Materials and Methods

The data were collected in the Study of Environment, Life-style & Fibroids (SELF), a prospective cohort study of fibroid development supported by the National Institute of Environmental Health Sciences, National Institutes of Health. SELF was designed to study risk factors for incident fibroids and fibroid growth. Given the marked difference in age of onset for fibroids by race[1], only women who self-identified as African American were invited to participate. The young age of onset in African Americans provides the opportunity to recruit young women with a high likelihood of developing fibroids over the course of the 5 year study. Premenopausal African American women, aged 23-34, who had not been diagnosed with fibroids were recruited between 2010 and 2012. Recruitment was designed to saturate the geographic area with information about the study (Detroit, Michigan and surrounding area). Materials included a website (detroitself.org), fliers, brochures at healthcare clinics, local radio, television, newspaper, and magazine advertisements, information booths at community events, and letters to women who had been seen by a doctor at Henry Ford Health Systems (HFHS), a large medical group in the Detroit area and collaborating institution. The letters were sent to women listed as 23-34 years old, with stratification by age to help maintain equal recruitment by age. Letters that encouraged recipients to tell others about the study were also sent to women older than the targeted age range. Recruitment techniques were designed to reach a representative sample of young African American women in Detroit and enroll volunteers who would be motivated to stay active in the cohort for at least 5 years. There is no estimate of the number of women who became aware of SELF, but census data indicate that 45,068 African American women age 23-34 were living in the City of Detroit in 2010[14]. 

Women who heard about the study and were interested in learning more phoned the study number, and were screened for eligibility (at least part African American or black; age 23-34 years old; no prior diagnosis of fibroids; no hysterectomy; never taken medication for lupus, Grave’s disease, scleroderma, or multiple sclerosis; no cancer requiring radiation or chemotherapy; live in U.S.; willing to attend study visits in Detroit over a 5-year period; and willing to provide information for tracing, i.e., social security number and/or contact information for persons who would know how to locate them). Eligible women with further interest received detailed information about the study during an orientation session. 

Women who chose to enroll after the orientation completed self-administered questionnaires, a telephone interview, and had a standardized research ultrasound examination to screen for the presence of fibroids. Interviews and questionnaires were all computer assisted and designed to prompt for further information following answers such as “Don’t Know” to minimize missing data. Women were not informed of ultrasound results prior to completion of baseline questionnaires, and ultrasound technicians were blinded to any collected baseline data.

Information on a history of abnormal scarring was obtained during the baseline telephone interview. As the distinction between hypertrophic scars and keloids can be difficult, women were asked both if they had “ever had a raised scar” and if they had ever had a “scar that grew up and out beyond the original injury”. Women who reported a raised scar but not a scar that grew beyond the original injury were assigned as hypertrophic scar. Women who reported a scar that grew beyond the original injury were assigned as keloid scar. Women who reported neither scar type were considered unaffected.

The ultrasound examination included a transvaginal scan with the addition of a transvesical/abdominal view if there was difficulty viewing the uterus with a transvaginal approach. In addition to noting the number and type (submucosal, intramural, subserosal, pedunculated) of any fibroids present, three perpendicular diameters (to nearest 0.1 cm) were measured for each fibroid. Each fibroid was measured in its three dimensions at three separate times during the examination, and the mean of each measure was used to calculate the volume (cubic centimeters, cc) of each fibroid using the ellipsoid formula (length*width*height*0.52). The maximum diameter (cm) for each fibroid was the maximum measurement recorded. The measurements of up to 6 fibroids were recorded for each woman. All measurements were inspected for outliers and questionable values were confirmed by the head sonographer using archived still and video images. A small number (N=4) of fibroids were not clearly visualized in all three planes for measurement. These fibroids were characterized as questionable and were excluded from the analysis of fibroid characteristics.

Covariate information was self-reported with the exception of measured height and weight. When measured height and weight were not available (N=8), self-reported values were used. Alcohol use was based on self-report of the time in her life when a woman drank “the most”. Women who never drank more than 10 drinks/year were classified as non-drinkers. Women who met the definition of binge drinking (>4 drinks per session at least twice a month) were classified as heavy drinkers. All other women were classified as moderate drinkers.

### Ethics Statement

Participants gave written informed consent, and the study was approved by the institutional review boards of the National Institute of Environmental Health Sciences and HFHS.

### Statistical Analysis

Given the reported difficulty in distinguishing between hypertrophic scars and keloids[7], abnormal scarring was considered as a group (raised scars+keloids) as well as distinct phenotypes. Initial descriptive analysis explored the distribution of demographic, anthropometric, reproductive and behavioral risk factors by scar type. 

Risk ratios with 95% confidence intervals were calculated using log linear regression models[15] for the binary outcome of presence of fibroids using three scarring phenotypes: hypertrophic (no keloid), keloid, and either scar type. Women who reported neither scar type were used as the reference group. As the association between keloids and fibroids is unlikely to be directly etiologic, the known fibroid risk factors[1] of age and parity at the time of the ultrasound examination were the sole *a priori* covariates. Categories of education also showed a possible association with scar type in the descriptive analysis and were included in the adjusted model. 

Among women with fibroids, specific characteristics of prevalent fibroids (type, number, largest diameter (cm) and total fibroid volume (cc)) were also examined with respect to scar type. Pearson chi-square tests and Mann Whitney U tests were used to assess differences between groups. Fisher’s exact tests were used in place of chi-square tests when expected cell counts were <5. SAS 9.3 (Cary, NC) was used for all analyses. As women could have more than one type of fibroid, differences by scar type were tested for each of 4 categories of fibroid type (submucosal, intramural, subserosal, pedunculated).

## Results

Baseline data collection for SELF was complete in early 2013; the cleaned data for both the questionnaires and the enrollment ultrasound are available for the first 1196 participants. Women had a mean age of 28.8 years (SD 3.4) and 78.5% had at least some education beyond high school. Household income was reflective of the Detroit geographic area[14] with 44.4% reporting a household income less than $20,000. Mean body mass index was 33.6 (kg/m^2^) (SD 9.5). Most women (72.7%) had experienced a pregnancy and 60.5% had given birth. 

Self-report of abnormal scars was common with 338 women (28.3%) reporting a raised scar and 108 (9.0%) reporting a keloid. Demographic, behavioral, reproductive and anthropometric factors did not vary by reported abnormal scarring with the exception of educational attainment which showed slightly more women with higher education reporting abnormal scars (Table 1). 

**Table 1:** Baseline Characteristics of Participants by Self-Reported Scar.

	No abnormal scarring	Raised Scar	Keloid	
Characteristic**^*a*^**	**N=750**	**N=338**	**N=108**	**p-value^*d*^**
Age				0.3
23-26 yrs	233 (31.1)	93 (27.5)	27 (25.0)	
27-30 yrs	262 (34.9)	109 (32.3)	39 (36.1)	
31-34 yrs	255 (34.0)	136 (40.2)	42 (38.9)	
Household Income**^*b*^**				0.2
<$20,000	351 (47.2)	129 (38.5)	51 (47.7)	
$20,000-$50,000	265 (35.6)	144 (43.0)	35 (32.7)	
>$50,000	128 (17.2)	62 (18.5)	21 (19.6)	
Education**^*c*^**				0.003
HS/GED or less	185 (24.7)	50 (14.8)	23 (21.3)	
Some college/Associates/Technical	363 (48.5)	171 (50.6)	51 (47.2)	
Bachelors/Masters/PhD	201 (26.8)	117 (34.6)	34 (31.5)	
Body Mass Index				0.06
<25 kg/m^2^	146 (19.5)	67 (19.8)	19 (17.6)	
25-29 kg/m^2^	161 (21.5)	50 (14.8)	28 (25.9)	
30-34 kg/m^2^	162 (21.6)	72 (21.3)	16 (14.8)	
>35 kg/m^2^	281 (37.5)	149 (44.1)	45 (41.7)	
Smoking History				0.2
Never smoked	559 (74.5)	252 (74.6)	71 (65.7)	
Former smoker	51 (6.8)	30 (8.9)	12 (11.1)	
Current smoker	140 (18.7)	56 (16.6)	25 (23.2)	
Alcohol Use				0.2
Non Drinker	207 (27.6)	78 (23.1)	21 (19.4)	
Moderate	245 (32.7)	125 (37.0)	37 (34.3)	
Heavy	298 (39.7)	135 (39.9)	50 (46.3)	
Menarche				0.3
<10 years	141 (18.8)	64 (18.9)	18 (16.7)	
11 years	151 (20.1)	64 (18.9)	16 (14.8)	
12 years	183 (24.4)	101 (29.9)	35 (32.4)	
13 years	120 (16.0)	48 (14.2)	22 (20.4)	
>14 years	155 (20.7)	61 (18.1)	17 (15.7)	
Reproductive History				0.2
Never Pregnant	206 (27.5)	98 (29.0)	22 (20.4)	
Nulliparous	93 (12.4)	39 (11.5)	14 (13.0)	
Parous: 1 birth	177 (23.6)	100 (29.6)	32 (29.6)	
Parous: 2 births	146 (19.5)	58 (17.2)	20 (18.5)	
Parous: 3+ births	128 (17.1)	43 (12.7)	20 (18.5)	

Abbreviations: *GED*, General Education Development; *SD*, standard deviation; yrs, years

***^a^***Data are presented as n (percentage) of individuals in each scar group. **^*b*^**Missing for Income: No Scar (N=6), Raised Scar (N=3), Keloid (N=1). **^*c*^**Missing for Education: No Scar (N=1). ***^d^***P-value for categorical comparisons using chi-square.

Fibroids were also a common finding with 279 women (23.3%) having at least one fibroid detected on ultrasound. Neither keloids nor hypertrophic scars were associated with the presence of fibroids with null associations for both distinct scar types as well as both scar types combined. Adjustment for age, education and parity, singly or as a group, did not materially impact the point estimates or precision (Table 2).

**Table 2:** Association [Risk Ratio (95% Confidence Interval) p-value] Between Self-Reported Scars and Prevalent Fibroids.

Scar Type	Fibroids [N (%)]	Unadjusted	Adjusted**^*a*^**
No abnormal Scarring	178 (23.7)	Ref	Ref
Any abnormal Scarring	101 (22.6)	0.95 (0.77-1.18) 0.7	0.89 (0.72-1.10) 0.3
Hypertrophic Scar	82 (24.3)	1.02 (0.81-1.28) 0.9	0.95 (0.75-1.18) 0.6
Keloid Scar	19 (17.6)	0.74 (0.48-1.14) 0.2	0.71 (0.47-1.08) 0.1

^a^ Adjusted for age category (23-26, 27-30, 31-34), education (high school or less, some education beyond high school, college degree) and parity (parous, nulliparous). Analysis for N=1195 due to one observation missing education.

Among the 275 women with distinct fibroids (Table 3), the majority were small (<2 cm in diameter) and intramural. Approximately 40% of the women had more than one fibroid documented. None of the fibroid characteristics was more strongly related to abnormal scarring than would be expected by chance, e.g women with large fibroids or fibroids in a particular uterine location were not more likely to have either hypertrophic scars or keloids.

**Table 3:** Characteristics of Prevalent Fibroids by Self-Reported Scar Status.

	**No abnormal scarring**	**Raised Scar**	**Keloid**	
**Fibroid Characteristics^*a*^**	N=176**^*b*^**	N=80**^*b*^**	N=19	p-value**^*c*^**
**Type^*d*^**				
**Submucosal**	18 (10.2)	13 (16.3)	0	0.1
**Intramural**	148 (84.1)	62 (77.5)	14 (73.7)	0.3
**Subserosal**	50 (28.4)	29 (36.3)	4 (21.1)	0.3
**Pedunculated**	6 (3.4)	4 (5.0)	2 (10.5)	0.2
**Max Diameter mean (SD)**	2.3 (1.8)	2.6 (1.8)	2.8 (2.7)	0.2
**<2 cm**	109 (61.9)	42 (52.5)	12 (63.2)	0.5
**2- 3.9 cm**	42 (23.9)	22 (27.5)	3 (15.8)	
**4+ cm**	25 (14.2)	16 (20.0)	4 (21.1)	
**Total Volume mean (SD)**	20.6 (66.5)	20.7 (43.0)	36.8 (93.3)**^*e*^**	0.2
**<1 cc**	61 (34.7)	22 (27.5)	8 (42.1)	0.5
**1- 4.9 cc**	61 (34.7)	25 (31.3)	5 (26.3)	
**5+ cc**	54 (30.7)	33 (41.3)	6 (31.6)	
**Number mean (SD)**	2.0 (1.6)	2.0 (1.5)	1.3 (0.6)	0.1
**Single**	99 (56.3)	49 (61.3)	15 (79.0)	0.2
**Multiple**	77 (43.8)	31 (38.8)	4 (21.1)	

***^a^***Data are presented as n (percentage) of fibroids within each scar group. ***^b^***N=4 women with questionable fibroids (N=2 in both No Scar and Raised Scar) not included. **^*c*^**Pearson Chi-Square tests for counts and Mann-Whitney U Test for continuous measures. Fisher’s exact test used when expected cell counts <5. **^*d*^**Women with multiple fibroids may have multiple types, so p-values reflect differences in percentages across scar categories within each type. **^*e*^**Includes one outlier, a woman with very large fibroids (380cc); mean Total Volume (SD) without this observation: 17.8 (43.6).

## Discussion

The intriguing similarities between keloids and fibroids have led to speculation that they may share similar etiologies. This is the first study, to our knowledge, to report the association between abnormal scarring and prevalent fibroids. The prevalence of both keloids and fibroids in this population is in keeping with previous studies[11,16]. The high prevalence of undiagnosed fibroids is not unexpected in this population. Screening studies to identify undiagnosed fibroids in young African American women have found similar prevalences[17,18]. While both abnormal scars and prevalent fibroids were relatively common in the SELF cohort, we found no evidence of an association between these two outcomes.

Apart from the increased prevalence of the two conditions in African Americans, the most compelling similarities between keloids and fibroids are the similarities in extracellular matrix[11] and the overexpression of TGFβ[3]. The lack of an association between the two conditions suggests that a susceptibility to fibrosis is probably not the main driver of their development. Differences in the precipitating events for these two outcomes may explain their lack of association. Although physical trauma is always an antecedent for keloids, the initiation of fibroids remains elusive.

Strengths of this study include the use of ultrasound to detect prevalent fibroids in a high risk population. We are limited however in the use of self-report for the presence of abnormal scars. Given the difficulty that clinicians have in distinguishing between keloids and hypertrophic scars[8], self-report of specific scar types is vulnerable to misclassification. Non-differential misclassification of the dichotomous scar exposure would make it more difficult to detect a true association[19].

In addition to the null association with the presence of fibroids, we also found no significant associations between distinct characteristics of the prevalent fibroids with scar type. Follow-up ultrasounds within this cohort will provide data on the growth trajectory of existing fibroids and the emergence of new fibroids. Any association with fibroid growth should be apparent after the 5 year follow-up is complete.

This study is the first to examine the association between keloids and fibroids in a large group of African American women at high risk of both outcomes. Although we find no association between self-reported keloids and ultrasound identified prevalent fibroids, on-going follow-up of this cohort will provide an opportunity to study possible associations with fibroid growth.
